# A multiphase study protocol of identifying, and predicting cancer-related symptom clusters: applying a mixed-method design and machine learning algorithms

**DOI:** 10.3389/fdgth.2024.1290689

**Published:** 2024-04-19

**Authors:** Mojtaba Miladinia, Kourosh Zarea, Mahin Gheibizadeh, Mina Jahangiri, Hossein Karimpourian, Darioush Rokhafroz

**Affiliations:** ^1^ Department of Nursing, School of Nursing and Midwifery, Ahvaz Jundishapur University of Medical Sciences, Ahvaz, Iran; ^2^ Nursing Care Research Center in Chronic Diseases, School of Nursing and Midwifery, Ahvaz Jundishapur University of Medical Sciences, Ahvaz, Iran; ^3^ Department of Biostatistics, Faculty of Medical Sciences, Tarbiat Modares University, Tehran, Iran; ^4^ Department of Medical Oncology, School of Medicine, Ahvaz Jundishapur University of Medical Sciences, Ahvaz, Iran

**Keywords:** nursing care, palliative care, qualitative research, quality of life, symptom management

## Abstract

**Objectives:**

In recent years, there has been increasing attention on the cluster approach to symptom management. Two significant challenges in the symptom cluster (SC) approach are identifying and predicting these clusters. This multiphase protocol aims to identify SCs in patients with advanced cancer as the primary objective, with the secondary objective of developing machine learning algorithms to predict SCs identified in the first phase.

**Methods:**

The 2-MIXIP study consists of two main phases. The first phase involves identifying SCs, and the second phase focuses on developing predictive algorithms for the identified SCs. The identification of SCs involves a parallel mixed-method design (quantitative and qualitative). Quantitative and qualitative methods are conducted simultaneously and given equal importance. The data are collected and analyzed independently before being integrated. The quantitative part is conducted using a descriptive-analytical method. The qualitative analysis is conducted using a content analysis approach. Then, the identified SCs from both parts are integrated to determine the final clusters and use them in the second phase. In the second phase, we employ a tree-based machine learning method to create predictive algorithms for SCs using key demographic and clinical patient characteristics.

**Conclusion:**

The findings of the 2-MIXIP study can help manage cancer patients' symptoms more effectively and enhance clinical decision-making by using SCs prediction. Furthermore, the results of this study can provide guidance for clinical trials aimed at managing symptoms.

## 1 Introduction

Patients with cancer typically experience multiple concurrent symptoms, which can make their management challenging and impact their quality of life (1). The term “symptom cluster” has been proposed to describe this phenomenon (2). This concept suggests that concurrent symptoms do not exist independently but interact with each other through mechanisms. A symptom cluster (SC) is defined as “two or more symptoms that appear simultaneously and are related to each other, which may have a common underlying cause or mechanism”. The connections between symptoms within a cluster are typically stronger than those between symptoms in other clusters (3, 4), and symptoms within a cluster mutually influence each other synergistically (5). A cluster of symptoms works cooperatively to increase patient suffering, and reduce treatment compliance, which may even affect patient survival compared with a single symptom. Consequently, in recent years, SC approach has received more attention in efforts to manage symptoms (6–8). SC research is an emerging field in symptom management that aims to enhance the understanding and treatment of cancer-related symptoms.

Identification of symptom clusters (SCs) is important for predicting other symptoms within a cluster, discovering possibly overlooked symptoms, making decisions to design an appropriate care plan, and individualizing interventions (9–11). There are two significant clinical advantages of SC approach. One reason is that when interventions are performed to improve a specific symptom in a cluster, other symptoms within that cluster may also be alleviated. In fact, by improving one symptom, the entire cluster can be improved. Another benefit of the patient care program is the ability to select interventions that address multiple symptoms within a cluster, rather than focusing on individual symptoms (8, 12, 13).

There are several challenges in the supply cluster approach. The primary challenge is to identify these clusters (8). Both quantitative and qualitative methods can be used for this purpose. With a quantitative approach, clusters can be identified using statistical clustering methods, although they may be subject to bias. The use of various definitions, diverse data collection tools, and different data analysis approaches has led to data sets that are challenging to interpret (8, 14). It is also important to note that current research focuses on clusters defined using statistical relationships between symptoms rather than those derived from patient experiences. An alternative approach to examining symptom clusters is through qualitative studies of symptom experience. Using a qualitative approach to identify SCs can overcome the limitations of statistical methods because it captures the unique experiences of patients (15, 16) and can lead to a deeper understanding of SCs from a patient-centered perspective rather than a statistics-based one. In fact, the use of both quantitative and qualitative methods can complement each other for a more comprehensive and accurate investigation of clusters. To our knowledge, no study has used the combination of both methods to identify SCs. The 2-MIXIP study is the first to simultaneously employ quantitative and qualitative methods to identify SCs in patients with advanced cancer. The second challenge is that the clusters of symptoms identified are obtained from a population of patients, and we need to determine how many clusters exist and what combinations are present in our target population. Until this point, they have more restricted clinical use. In a real clinical setting, identifying the cluster(s) our patient belongs to is a very challenging, time-consuming, and complex task that can sometimes exceed the capabilities of nurses and the healthcare team. Furthermore, the patient's condition may change at any moment, including changes in the disease stage, severity of symptoms, and so on, which can result in alterations to the cluster's condition, making continuous assessments unfeasible. Therefore, to address this clinical gap, the method of predicting SCs can be used. However, the main challenge in this field is to use methods that yield the highest prediction accuracy (8). One of the most powerful methods is machine learning techniques (17). To the best of our knowledge, no study has been conducted to predict cancer-related symptom clusters using advanced methods.

### 1.1 Objectives

According to the challenges mentioned, the primary goal of the 2-MIXIP multiphase study is to identify SCs in patients with advanced cancer undergoing active treatment. The secondary objective is to develop machine learning algorithms to predict symptom clusters identified in the initial phase. This makes the clinical application of these clusters in symptom management more practical for nurses and palliative care teams.

## 2 Method

### 2.1 Design and setting

**Figure 1:**
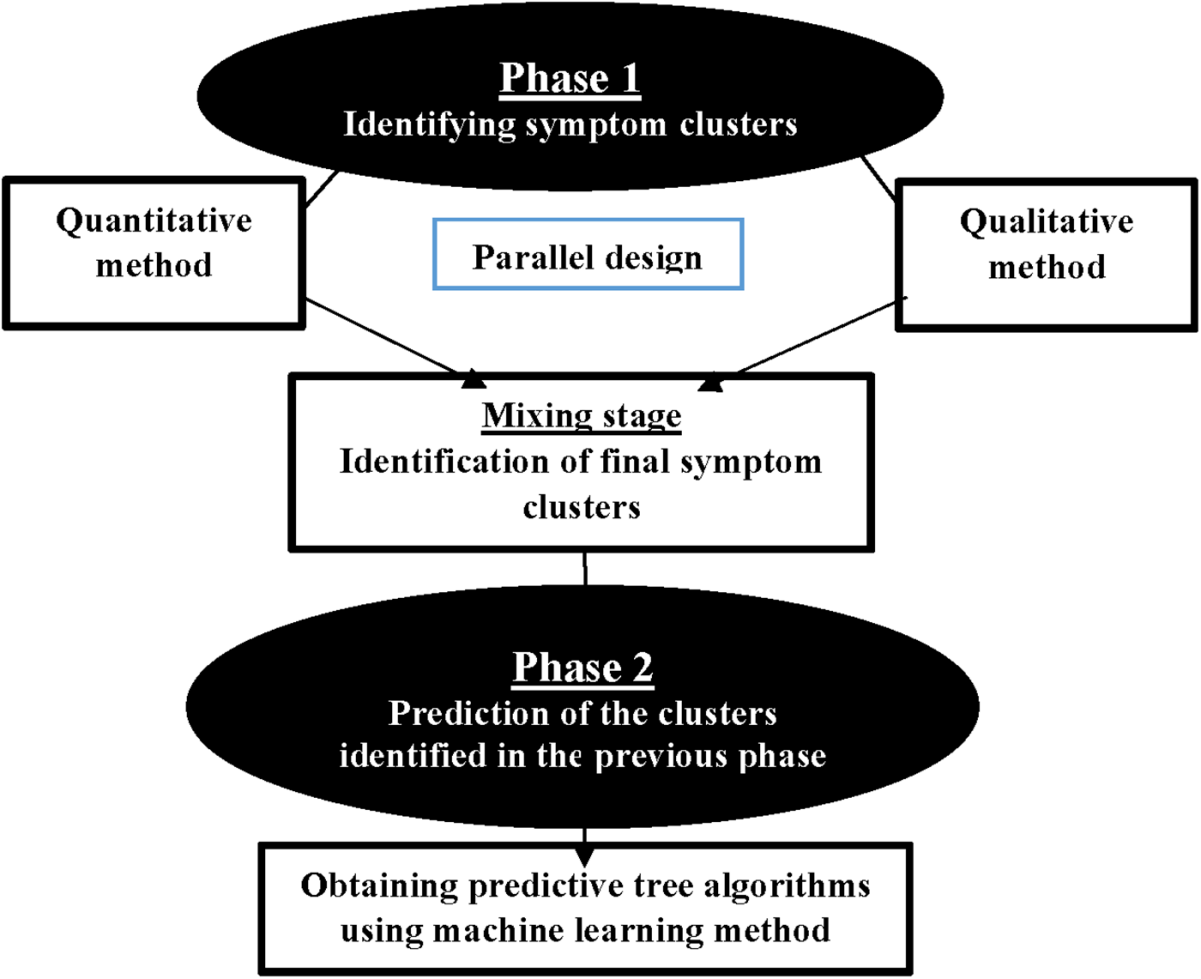
Study phases.

This study employs a multiphase design to investigate advanced cancer in adults. It consists of two main phases (see Figure 1). The first phase involves identifying SCs, whereas the second phase focuses on developing predictive algorithms for the identified SCs using machine learning methods. The study is conducted at three academic centers: Baghai 2 Teaching Hospital, the Clinical Oncology Department of Golestan Hospital, and Shafa Oncology Clinic, all affiliated with Ahvaz Jundishapur University of Medical Sciences (AJUMS), Ahvaz, Iran. This study has been approved by the Institutional Review Board affiliated with AJUMS (IR.AJUMS.REC.1402.046). Written informed consent is obtained from all participants.

### 2.2 Phase 1 (identifying symptom clusters)

The main objective of the initial phase of the study is to identify symptom clusters. The identification of clusters is conducted using a parallel mixed-method design, incorporating both quantitative and qualitative approaches. Two quantitative and qualitative stages are conducted simultaneously and given equal importance. The data are collected and analyzed independently before being combined.

#### 2.2.1 Quantitative stage

The quantitative stage is conducted using a descriptive-analytical method. A convenience sampling method is used in the quantitative phase. The inclusion criteria comprised the following: (1) being in an advanced stage of cancer; (2) undergoing active treatment; (3) adults aged 18 years and older; and (4) ability to communicate effectively. Exclusion criteria include: (1) a history of mental illness or cognitive impairment; (2) unstable clinical condition; (3) concurrent serious medical conditions.

Quantitative Data Collection: Data are collected using a demographic characteristics form and the Memorial Symptom Assessment Scale (MSAS) questionnaire. The MSAS is a self-report questionnaire designed by Portenoy et al. in 1994 to measure multidimensional symptom experience. The MSAS contains 32 common symptoms divided into two parts. The first part contains 24 symptoms, including three aspects of occurrence (frequency), intensity, and distress for each symptom. The second part includes 8 symptoms that are assessed solely based on their intensity and distress. Using the MSAS, patients are asked to indicate whether they have experienced any of the symptoms in the past week (i.e., symptom occurrence). If individuals have experienced these symptoms, they are asked to assess their frequency, intensity, and distress. The MSAS is scored using the Likert scale. It also includes an open-ended question at the end that asks patient to list any additional symptoms beyond the 32 specified (18). The reliability and validity of the MSAS have been well established in oncology patients (19–22). In Bahrami et al.'s study, the MSAS demonstrated good validity and reliability among cancer patients in Iran. The Cronbach's alpha coefficient was 0.77 (*α* = 0.77) (23).

##### 2.2.1.1 Sample size

In factor analysis studies with numerous variables, one method for estimating the sample size is to apply a general rule (rule of thumb), which suggests that a minimum of 3–20 subjects per item should be considered necessary to avoid calculation problems (24–28). As the MSAS examines 32 symptoms in general, with 20 samples considered for each item, the estimated sample size for the quantitative stage of this study is 640 people.

Quantitative Data Analysis: Data are analyzed using R software Version 4.2.2. Descriptive statistics are presented as frequency/percentage, as well as mean and standard deviation (SD). One powerful method that can be used to identify clusters of symptoms is network analysis (NA). In general, networks are defined as a set of interconnected components, such as symptoms in this research. NA is a graph-based method that can identify the relationships between symptoms and various clusters of symptoms experienced by patients, and visualize and interpret them quantitatively. Network analysis (NA) can be helpful in identifying key symptoms that affect other concurrent symptoms or clusters, which are potential targets for therapeutic interventions (19). The primary function, estimateNetwork, employs the least absolute shrinkage and selection operator (LASSO) with extended Bayesian information criterion (EBIC) model selection, utilizing a hypertuning parameter (*γ*) to determine model sparsity. Polychoric correlations are computed for ordinal data, establishing a Gaussian graphical model with nodes representing items and edges denoting partial correlations. Four centrality indices (strength, betweenness, closeness, and expected influence) are identified as pivotal nodes in the network. The study also explores bridge nodes connecting different communities, using bridge-expected influence metrics to identify nodes likely to activate nearby communities. Overall, this study employs advanced network estimation techniques, centrality indices, and bridge node analysis to comprehensively investigate the interconnectedness within the network.

#### 2.2.2 Qualitative stage

In this stage, the qualitative method of content analysis is used to identify SCs, and it is conducted simultaneously with the quantitative stage. The participants for the qualitative phase are selected from the participants in the quantitative phase who experience three or more co-occurring symptoms upon entering the study and are willing to share their experiences. In the qualitative stage, the purposive sampling method is used. Also, we will strive to achieve maximum diversity in our sampling to capture a wide range of experiences.

##### 2.2.2.1 Sample size

Sampling continues until data saturation is reached. Data saturation occurs when no additional data are available to further expand the information. The criterion for reaching this stage is the repetition of previous data, ensuring that the researcher consistently encounters data that confirms the previous findings. This process aims to ensure that the arrival of new people does not alter the researcher's decision and final summary. At this stage, the researcher concludes the selection of new participants (29).

##### 2.2.2.2 Qualitative data collection

When conducting research, the researcher identifies suitable participants and obtains their consent to participate. The time and place of the interview are then determined. The data collection method involves individual face-to-face semi-structured interviews. The interview process follows a general guide and begins with a broad question, such as “How have you been feeling physically in the past week?” This is followed by questions about the psychological symptoms experienced by the patient and how these symptoms relate to their physical experiences. After addressing these broad questions, the interview process continues with probing questions. Participants' experiences of symptoms are thoroughly explored in the interviews, and each mention of multiple symptoms is examined in greater detail. Participants are not asked about specific symptoms, as the interview questions are broad enough to capture important aspects of each participant's symptom experience. During the interview, probing and clarifying questions such as “Can you explain more?” were asked. “Can you give an example?” etc. are done for further investigation. Any new issues identified during the initial interviews are incorporated into the interview guide for subsequent interviews. In addition, field notes and reflexive reports are maintained after each interview to aid subsequent interviews and data analysis.

The interview is conducted in a location with appropriate environmental conditions. The main factors that determine the duration of the interview include the interviewee's tolerance, the volume of information, and the willingness of the participants. Multiple interviews may be conducted for each participant if necessary, as they facilitate in-depth reflection for both the participant and the researcher throughout the data collection process. All interviews are recorded with the participants' consent, and they are assured of the confidentiality of the information and restricted access to the recorded voice. Noted that participants have the option to decline audio recording of their speech. In such cases, the audio recorder device is turned off, and the required information is documented in writing. After each interview, the recorded material is transcribed verbatim into text.

##### 2.2.2.3 Qualitative data analysis

Qualitative data are analyzed concurrently with data collection using conventional content analysis, following the steps outlined by Elo and Kyngas (30). Data analysis ideally begins after the first interview and continues throughout the research process. This iterative process of moving back and forth between data collection and analysis enables researchers to explore relevant concepts and their dimensions (31). Analyzing data during the initial stages of collection can guide subsequent data collection to obtain relevant information (32). The categories are compared by two researchers, as well as the project's lead researcher. The analyzed categories are compared and discussed until a consensus is reached. If more than two participants independently mentioned an association between at least two symptoms, they were grouped together. Quotations are provided for each code and category of data in preparation for reporting findings. Depending on the study's purpose, researchers may opt to identify relationships between categories and subcategories based on their concurrence, antecedents, or consequences. Additionally, MAXQDA software is used for data management.

##### 2.2.2.4 Trustworthiness of qualitative data

The trustworthiness and authenticity of qualitative data are evaluated based on the criteria outlined by Lincoln and Guba (33–35). To ensure confirmability, the research team reviews the data, acting as a peer review process to verify that the analyzed data represent true findings and are free from potential bias. Additionally, the method of returning the data to the participants and obtaining their approval are used, known as member checking. The reliability of the research is determined by the researcher's consistent involvement with the research data, dedicating sufficient time collecting the data, checking the extracted data, and analyzing them. All interviews are recorded, and exact quotes from participants are provided to illustrate identified categories, thereby reducing the risk of researchers selectively filtering data through recall or summarization and ensuring greater reliability. Credibility is achieved through a wide variety of samples. To enhance validity, the identified SCs are reviewed by a specialized group comprising an oncologist, an oncology nurse, and the research team. They use their clinical experiences to review and confirm the data. Also, a group meeting is held with several patients to discuss the identified SCs and compare them with their experiences once again. To enhance replicability, all stages of research, particularly data analysis and category formation, are thoroughly documented. This allows other researchers to review the available documentation in the respective field. The researcher thoroughly describes the features and characteristics of the participants, and the findings are presented in the form of articles at international conferences.

#### 2.2.3 Mixing/integration stage

In this stage, the findings from both the quantitative and qualitative parts are integrated. The identified SCs from both parts are combined for use in the second phase of the study, which involves predicting clusters. In this study, we adhere to Creswell's approach (36). Creswell argues that the parallel combination method is the most suitable approach for comparing different perspectives derived from quantitative and qualitative data. The expected outcome involves integrating two databases to demonstrate how the data converge or diverge (Creswell, 53). In this study, quantitative and qualitative methods complement each other, with each method identifying clusters based on its strengths to provide a more comprehensive view of symptom clusters. In the first step of integration, the identified clusters are placed against each other. In the second step, we compare the two to identify similarities and differences. In the third and final step, we determine the ultimate clusters by identifying the number of clusters and the symptoms contained in each cluster. During the integration stage of SCs, we encounter three modes. Table 1 displays the modes and the research team's approach.

**Table 1:** Integration stage of qualitative and quantitative data.

Possible modes	Approach
Symptom clusters identified using both quantitative and qualitative methods are similar and identical.	Confirm with each other
A symptom cluster identified in the quantitative method may not be found in the qualitative method, and vice versa.	Clusters are combined, resulting in the creation of even more clusters.
In each method, a specific symptom should be placed in a separate cluster, and there should be no overlap between the two methods.	A specialized panel, comprising an oncologist, an experienced oncology nurse, members of the research team, and, if necessary, several patients, will be formed. Based on their clinical experience and data review, this panel determines the cluster in which this symptom will be categorized.

### 2.3 Phase 2 (predicting symptom clusters)

The main objective of the second phase is to predict SCs identified in the first phase of the study. In this phase, we use machine learning (ML) methods to develop predictive algorithms for SCs based on key demographic and clinical characteristics such as age, sex, type of cancer, and type of treatment. Machine learning encompasses various techniques. In this study, we focus on tree-based machine learning algorithms (decision trees) to predict clusters within different patient subgroups.

To develop predictive machine learning algorithms, various steps must be taken. The main steps include (37): (1) Training: a process during which the system is provided with both the input data and the correct outcome (answer) to learn from initially. (2) Testing: To evaluate the performance of trained algorithms, it is essential to compare the predictions made by the algorithm on a separate dataset from the one used for training, with the actual results for that dataset, which were known but not disclosed during training. (3) Tuning: The process of optimizing the parameters that affect the model to enable the algorithm to perform at its best. (4) Determining the accuracy, sensitivity, and specificity of the predictive algorithm. (5) Production of the final algorithm.

For prediction, we input important demographic and clinical variables as predictor variables, and SCs as the outcome, into the model. This allows the prediction algorithm to be generated based on these variables for homogeneous subgroups of patients. The samples in the second phase of the study are identical to those in the first phase. As a result, 70% of the samples from the first phase are used for machine training, while the remaining 30% will be used for testing. The R programing software is used to generate the algorithm.

## 3 Discussion

The 2-MIXIP study consists of two main phases: identifying and predicting cancer-related symptom clusters. In the first phase, we use both quantitative and qualitative approaches simultaneously to identify clusters. Using a qualitative approach to identify symptom clusters can offer valuable insights into the types of clusters that patients experience, as well as uncover clusters that may go undetected by statistical methods. Additionally, the qualitative approach can help prioritize the clusters based on their significance for patients and identify the predominant symptom in each cluster that holds great clinical importance. Qualitative methods may provide valuable data on how patients perceive, prioritize, and evaluate symptom clusters, which may aid in making decisions about more effective symptom management (2). In fact, the qualitative method provides an opportunity to explore the breadth and complexity of related symptoms. Qualitative methods have been used in fewer studies than quantitative methods to examine SC in oncology patients (16, 38). More studies with a qualitative approach are needed to comprehensively understand SC in this patient population.

In the second phase of the study, tree-based machine learning algorithms are used to predict the identified SCs from the first phase. Tree-based algorithms enable the creation of predictive models that offer accuracy, stability, and ease of interpretation. Unlike linear models, nonlinear models depict nonlinear relationships well and can simultaneously consider several variables for prediction. Tree algorithms can handle both categorical and continuous variables (39). Although the process of generating these tree algorithms is complex, they are actually very simple to understand. Machine learning algorithms can greatly enhance prediction accuracy compared with conventional statistical regression models by capturing complex and nonlinear relationships in the data. In general, decision trees offer several advantages, including easy understanding, powerful data exploration, minimal need for data cleaning, resilience to outliers and missing values, no requirement for data normalization during processing, and no restrictions on data type (40, 41).

The implementation of this study faces several limitations. This study also has several limitations. Participation is limited to patients who speak Farsi. It is not possible to interview patients who experience numerous symptoms but are in a serious physical condition. The variables are subjective in nature, which makes them vulnerable to the influence of cultural differences, individual variations, and family, social, and personal challenges. Additionally, this study was only conducted in one city, so it may not account for cultural, social, racial differences, etc., which could impact the generalizability of the results to some extent.

## 4 Conclusion

The findings of the 2-MIXIP study help to effectively manage symptoms in patients with advanced cancer, thereby improving their quality of life. The clinical decision-making authority of the care team, particularly nurses, is enhanced by predicting symptom clusters. This enables them to select more effective interventions that address a group of symptoms rather than a single symptom. Identifying SCs can also help individualize interventions (10, 11). Furthermore, the results of this study serve as the foundation for future research, such as clinical trials for managing symptoms and the creation of clinical instruments for evaluating clusters of cancer-related symptoms.

## Data Availability

The final dataset and the code for statistical analysis will be available upon reasonable request.
